# New insights into microbial bile salt hydrolases: from physiological roles to potential applications

**DOI:** 10.3389/fmicb.2025.1513541

**Published:** 2025-02-12

**Authors:** Zixing Dong, Shuangshuang Yang, Cunduo Tang, Dandan Li, Yunchao Kan, Lunguang Yao

**Affiliations:** ^1^Henan Province Engineering Research Center of Insect Bioreactor, College of Life Sciences, Nanyang Normal University, Nanyang, China; ^2^College of Physical Education, Nanyang Normal University, Nanyang, China; ^3^China-UK-NYNU-RRes Joint Laboratory of Insect Biology, Henan Key Laboratory of Insect Biology in Funiu Mountain, Nanyang Normal University, Nanyang, China

**Keywords:** gut microbiota, host health, bile acids, bile salt hydrolase, physiological roles, potential applications

## Abstract

Gut microbiota has been increasingly linked to metabolic health and diseases over the past few decades. Bile acids (BAs), the major components of bile, are bidirectionally linked to intestinal microbiota, also known as the gut microbiome-BA metabolic axis. Gut microbiota-derived bile salt hydrolase (BSH, EC 3.5.1.24), which catalyzes the “gateway” reaction in a wider pathway of bile acid modification, not only shapes the bile acid landscape, but also modulates the crosstalk between gut microbiota and host health. Therefore, microbial BSHs exhibit the potential to directly or indirectly influence microbial and host physiologies, and have been increasingly considered as promising targets for the modulation of gut microbiota to benefit animal and human health. However, their physiological functions in bacterial and host physiologies are still controversial and not clear. In this review, we mainly discuss the current evidence related to the physiological roles that BSHs played in gut microbiota and human health, and the possible underlying mechanisms. Meanwhile, we also present the potential applications of BSHs and BSH-producing probiotics in various fields. Finally, we describe several important questions that need to be addressed by further investigations. A detailed exploration of the physiological significance of BSHs will contribute to their future diagnostic and therapeutic applications in improving animal and human health.

## 1 Introduction

Gut microbiota is a large population of commensal microorganisms inhabiting the gastrointestinal tract (GIT), including bacteria, viruses, archaea, protozoa and fungi (Parasar et al., 2019). Due to its ability to affect intestinal permeability, motility and sensitivity, mucosal immune function, and enteric nervous system, gut microbiome has been shown to play crucial roles in host health, while disruption of this population has been implicated in many diseases (Foley et al., 2021). For this reason, modulation of gut microbiota composition has become a potential alternative approach for preventing and treating diseases to maintain human health.

**Figure 1:**
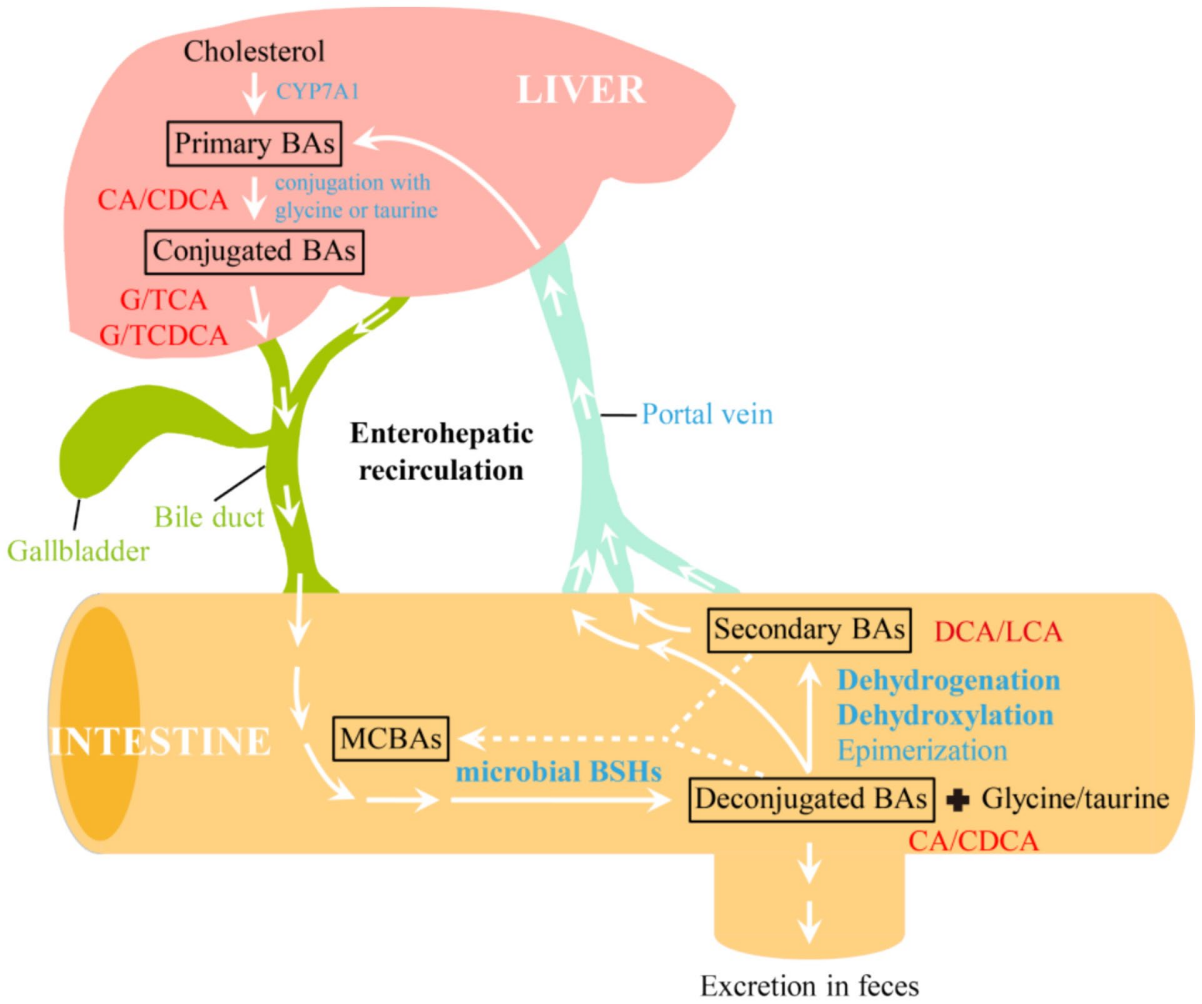
Enterohepatic circulation and modifications of bile acids by gut microbiome. CYP7A1, cholesterol 7α-hydroxylase; BSHs, bile salt hydrolases; BAs, bile acids; MCBAs, microbial conjugated bile acids; CA, cholic acid; CDCA, chenodeoxycholic acid; G/TCA, glyco- and taurocholic acids; G/TCDCA, glyco- and taurochenodeoxycholic acids; DCA, deoxycholic acid; LCA, lithocholic acid.

As the major components of bile, bile acids (BAs) represent an important class of metabolites that shape the gut microbiota, host physiology and metabolism. After *de novo* synthesis from cholesterol by host hepatocytes through cholesterol 7α-hydroxylase (CYP7A1), primary BAs, such as cholic acid (CA) and chenodeoxycholic acid (CDCA), are conjugated with either glycine or taurine to form conjugated bile acids, including glyco- and taurocholic acids (G/TCA) as well as glyco- and taurochenodeoxycholic acids (G/TCDCA, Figure 1). During the fasting state, these conjugated BAs are stored in the gallbladder. Upon ingestion of foods, they are excreted into the proximal small intestine via bile duct to promote the emulsification and absorption of dietary lipids and lipophilic vitamins (Yang et al., 2022). As they travel through the small intestine, the majority of conjugated BAs (>95%) are deconjugated by gut microbiota, and absorbed by active transport. The unabsorbed deconjugated BAs (CA or CDCA) are converted into secondary BAs [deoxycholic acid (DCA), lithocholic acid (LCA), etc.] through further microbial modifications, and absorbed into colon via passive diffusion. Both deconjugated and secondary BAs reabsorbed from the gut are taken up by hepatocytes through portal vein, reconjugated, and resecreted into bile for enterohepatic recirculation (Chiang and Ferrell, 2022), whereas remaining BAs are excreted into the feces. Recently, it has been found that BSHs from *Lactiplantibacillus* strains (Foley et al., 2023), *B. longum* NCTC 11818 (Rimal et al., 2024) and *C. perfringens* (Guzior et al., 2024) also exhibit amine *N*-acyl transferase activity that mediates the conjugation of amino acids to the acyl-site of deconjugated and secondary BAs, generating a diverse repertoire of microbial conjugated bile acids (MCBAs). This newly found function of BSHs expands BA diversity and complexity, and the compounds obtained also serve as substrates for BSHs and enter enterohepatic recirculation.

**Figure 2:**
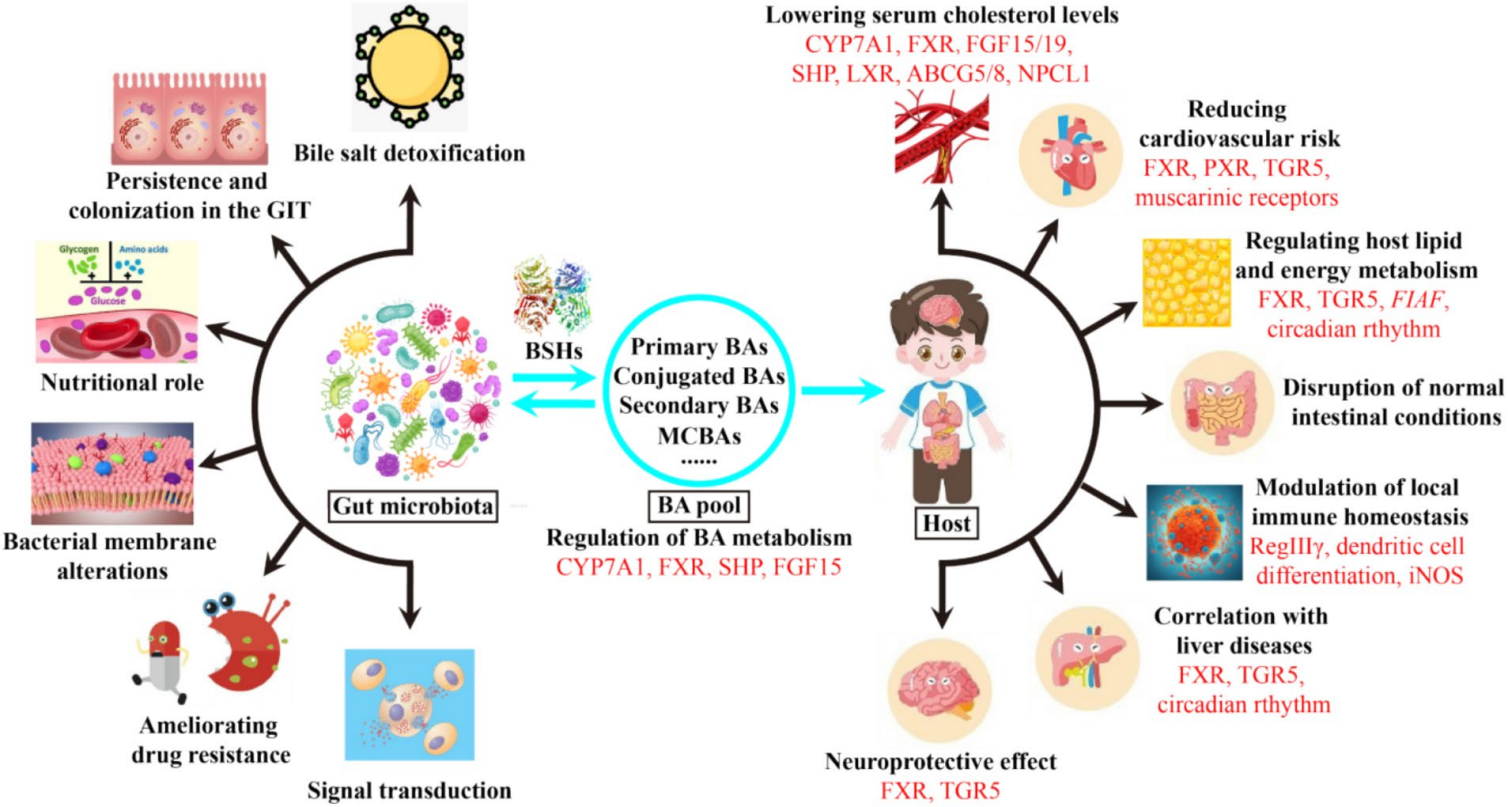
Effects of microbial BSHs on the bacterial and host physiologies. Bile acid receptors and related genes involved in each physiology are marked in red. GIT, gastrointestinal tract; MCBAs, microbial conjugated bile acids; FXR, farnesoid X receptor; FGF15/19, fibroblast growth factors 15 and 19; SHP, small heterodimer partner; LXR, liver X receptor; ABCG5/8, adenosine triphosphate-binding cassette transporters G5 and G8; NPCL1, Niemann-Pick C1-like 1; PXR, pregnane X receptor; TGR5, G protein-coupled bile acid receptor 1; *FIAF*, fasting-induced adipose factor; RegIIIγ, antimicrobial peptide RegIIIγ; iNOS, inducible nitric oxide synthase.

Intestinal microbiota and BAs are bidirectionally linked to form the well-known gut microbiome-BA metabolic axis (Figure 2), which has been increasingly recognized as a key regulator of host health (Wang et al., 2022). On the one hand, gut microbiota have evolved many strategies to modify BAs and change the BA pool, which in turn regulates BA metabolism by receptors and promotes bacterial survival (Guzior and Quinn, 2021). On the other hand, the amphipathic nature of BAs confer them detergent-like antimicrobial activities that inherently restrict the growth of some bacteria, thus reshaping the composition of gut microbiota (Peng et al., 2024). As metabolic integrators, BAs can also bind to a variety of nuclear and cell-surface receptors in enterocytes, liver and adipose tissues, including nuclear receptor farnesoid X receptor (FXR), liver X receptor (LXR), pregnane X receptor (PXR) or the G protein-coupled bile acid receptor 1 (GPBAR1, also known as TGR5) (Mohanty et al., 2024b; Zhang et al., 2024). By acting as agonists or antagonists for these receptors, BAs can regulate their own synthesis, immune homeostasis (Liu et al., 2022; Mohanty et al., 2024a), metabolic syndromes (Wang N. et al., 2021), lipid and energy metabolism (Govindarajan et al., 2016; Huang et al., 2019), cholesterol metabolism (Samson et al., 2024), neurological disorders (Sun et al., 2022; Wu et al., 2024), and drug absorption and bioavailability (Enright et al., 2018) (Figure 2). Therefore, bile acids act as key mediators to modulate the communications between gut microbiota and host health.

Currently known microbial modifications of BAs include deconjugation, dehydroxylation, epimerization, and dehydrogenation (Figure 1) (Guzior and Quinn, 2021; Mohanty et al., 2024b). Among them, deconjugation reaction which hydrolyzes conjugated BAs into less toxic unconjugated BAs and amino acids is considered as a “gatekeeper” for subsequent microbial modifications of BAs. This reaction is catalyzed by bile salt hydrolase (BSH, EC 3.5.1.24), an enzyme belonging to the N-terminal nucleophilic (Ntn) hydrolase superfamily. There are now more than 2,400 BSH enzymes widely distributed among gut-associated bacterial phyla inhabiting both the small intestine and large bowel of human, including *Firmicutes*, *Bacteroidetes*, and *Actinobacteria* (Jones et al., 2008; Mohanty et al., 2024a). It has been reported that *Lactobacilli* strains, which constitute an important part of the *Firmicutes* bacteria, are the main contributors to total BSH activity in the intestinal tracts of human (Song et al., 2023) and that they help to modulate the enterohepatic circulation. Gut microbial BSHs are therefore highly required for the formation and upkeep of the BA pool, and BSH-mediated BA metabolism modulates a microbe-host dialog that alters both local (gastrointestinal) and systematic (hepatic) host functions, including shaping gut microbiota (Govindarajan et al., 2016; Lynch et al., 2023) and regulation of host metabolism (Lin et al., 2014; Miremadi et al., 2014). Thus, gut microbiota-derived BSHs have become promising microbiome targets for the development of novel therapies to prevent and treat human diseases.

However, it is somewhat controversial whether the BSH activity of gut microbiome is beneficial or detrimental to the host. Although the potential positive aspects of BSH enzymes have been previously discussed (De Smet et al., 1998), other possible negative concerns about them have also been raised, such as disruption of lipid metabolism, colon carcinogenesis, gallstone formation, altered gut microbial populations and other gut associated disorders (Jones et al., 2008). We review here the available literature on the physiological roles of BSH activity for the enzyme-producing bacteria as well as for the hosts, including the contradictory findings obtained and the underlying mechanisms involved. We also present the potential applications of BSHs and BSH-harboring probiotics. Finally, we conclude with suggestions for future work. Knowledge gained here can provide an updated understanding of the physiological significance of microbial BSHs and support their wide applications in BSH-based biotherapeutics development.

## 2 Physiological roles of microbial BSHs

Although the physiological roles of microbial BSHs are not yet fully understood, and may vary between bacterial species and genera, they have now been hypothesized to play multiple roles in the bacteria and hosts through several possible mechanisms, which are described in Figure 2 and briefly discussed below. Besides, they also have several potential applications in medical diagnosis, prevention and treatment of metabolic diseases, feed additives and other industries. Moreover, research is still ongoing to discern their exact functions.

### 2.1 Functions of BSHs in bacterial physiology

#### 2.1.1 Bile salt detoxification

Due to the lipophilic nature of the steroid ring, BAs are able to disorganize the cell membrane structure, acidify the cytoplasm and trigger DNA damage, displaying potent antimicrobial activities (Bustos et al., 2018). Therefore, bile represents a major challenge for bacteria to transit and survive in the GIT. Since BSH can convert more toxic conjugated BAs into less toxic free BAs, it has become a flexible response of gut microbes to BA stress and a desirable property when selecting a strain for use as a dietary adjunct (Urdaneta and Casadesús, 2017). In bacterial pathogens like *Listeria innocua* (Jones et al., 2008), *L. monocytogenes* (Begley et al., 2005) and *Brucella abortus* (Delpino et al., 2007), BSHs confer them with the ability to withstand the antimicrobial action of BAs in the gut, thus constituting a virulence factor for these bacteria, while *bsh* gene repression or disruption impairs the bile resistance of *L. monocytogenes* (Pombinho et al., 2020). Similarly, several studies have also established a connection between bile salt hydrolysis and bile tolerance of probiotic strains. For example, BSHs from *Lactiplantibacillus plantarum* WCFS1 (Lambert et al., 2008), *L. gasseri* JCM1131^T^ (Kusada et al., 2021), *Leptogranulimonas caecicola* TOC12T and *Granulimonas faecalis* OPF53T (Morinaga et al., 2022) confer bile resistance on these bacteria, and the expression of *bsh* genes from *L. plantarum* Lp91 (Duary et al., 2012), *B. longum* NCC2705 (Yuan et al., 2008) and *L. reuteri* CRL1098 (Bustos et al., 2015) was also up-regulated in the presence of bile salts, whereas disruption of *bsh* genes in *L. plantarum* WCFS1 (Lambert et al., 2008), *L. amylovorous* (Grill et al., 2000), *L. plantarum* AR113 (Wang G. et al., 2021), and *L. rhamnosus* AMC010 (Arnold et al., 2018) renders these strains more susceptible to bile salts. Furthermore, several *bsh* genes have been heterologously expressed in BSH-negative strains *L. innocua* FH2333 (Jones et al., 2008), *L. casei* HX01 (Wang et al., 2011) and *Leuconostoc citreum* CB2567 (Seung Kee et al., 2015), which improves their survivability under bile stress or gut colonization in mice, contributing to their probiotic properties. Together, these findings lay the groundwork for engineering *Lactobacilli* with improved bile resistance to be used as antimicrobial agents or BA-altering therapeutics.

However, previous studies have demonstrated that bile tolerance of probiotics is not necessarily linearly dependent on their BSH activity, some of which have even failed to provide a direct link between them (Moser and Savage, 2001; Fang et al., 2009). BSHs from some *Lactobacilli* strains have been shown to confer no bile protection on them (Yang et al., 2019; Foley et al., 2021), and that *L. monocytogenes* relies upon other biomolecules rather than BSH activity for its growth in the murine gall bladder (Dowd et al., 2011). These conflicting findings can be partially attributed to the different concentrations, types and structures of BAs used in various studies, which determine their toxicity and differences in BSH substrate specificity (Bustos et al., 2018). It has been shown that the toxicity of glycoconjugated BAs is far greater than that of their tauroconjugated counterparts, and this may be the reason why most BSHs prefer to hydrolyze the former (Begley et al., 2005; Dong and Lee, 2018). Besides, intrinsic bile resistance is also a strain-specific trait and cannot be generalized within a species or a genus (Li and Chiang, 2014; Bustos et al., 2018). Given the complex and multifactorial effects of BA deconjugation, it is possible that such discrepancies are due to the use of different types of BAs, BSHs with various substrate specificities, and different strains and species among the studies.

Although the precise physiological function of BSHs in assisting probiotics to overcome the toxicity of conjugated BAs remains poorly understood, several possible mechanisms evolved by BSH-producing microorganisms have been proposed. Some studies have suggested that BSHs comprise part of a global response to acid and bile stresses (Dimarzio, 2016). Protonated forms of conjugated BAs are organic acids that exhibit toxicity toward the bacteria through intracellular acidification (Grill et al., 2000), while the unconjugated BAs released by BSHs are weaker acids and less soluble than their conjugated counterparts, thus recapturing the co-transported proton which may counteract the drop in internal pH under bile salts (Bustos et al., 2012). Unconjugated BAs produced by BSH hydrolysis can form biofilms on the cell surface under bile stress, which is another important bile resistance mechanism in several strains, including *Lactiplantibacillus* (Ambalam et al., 2012), *Bifidobacteria* (Jarocki et al., 2014), and *Bacteroides fragilis* (Pumbwe et al., 2007). Besides, BSH activity is also part of a broader strategy to alter cell membrane composition in response to environmental stress. In *L. reuteri* CRL 1098, BSHs change the fatty acid composition of the cell membrane, which can form complexes with precipitated BAs and cholesterol to maintain membrane fluidity, thus providing bile tolerance (Taranto et al., 2003). Recently, heterologous expression of the *bsh* gene from *L. salivarius* in *Lactococcus lactis* NZ9000 revealed that unconjugated BAs could form micelles around membrane surface of cells to combat the toxicity of conjugated BAs (Bi et al., 2016). Apart from the ability to deconjugated BAs, there are also other mechanisms involved in the bacterial bile tolerance, like PrfA-regulated bile exclusion system (Sleator et al., 2005) and Sigma B-regulated detoxification mechanism (Dowd et al., 2011) in *L. monocytogenes*, surface proteins, fatty acids and exopolysaccharides (Horáčková et al., 2018).

#### 2.1.2 Persistence and colonization in the GIT

Adhesive property is an important factor that contributes to the colonization and survival of probiotics in the host gut, thus helping them exert health-promoting effects (Wu et al., 2022). For this reason, the ability to adhere to GIT has been proposed as one of the criteria for the selection of new probiotic strains. As BSHs can combat the deleterious effects of bile, BSH-mediated BA metabolism is an important function to assist microbial survival, gut persistence and colonization in the GIT (Pumbwe et al., 2007; Joyce et al., 2014b; Prete et al., 2020). In some pathogens, including *Enterococcus faecalis* (Shankar et al., 2002), *L. monocytogenes* (Begley et al., 2005) and *B. abortus* (Delpino et al., 2007), BSH activity has been identified as a virulence factor that influences bacterial persistence and dissemination in the host intestine. Accumulating evidence also suggests that BSHs improve mucosal adhesion and colonization abilities of probiotic strains in the GIT (Núñez-Sánchez et al., 2022), such as *L. reuteri* (Rodes et al., 2013), *L. plantarum* (Yang et al., 2019) and *L. plantarum* AR113 (Wu et al., 2022), further highlighting their significance as colonization factors (Jones et al., 2008; Grimm et al., 2014). Heterologous expression of *bshA* from *L. acidophilus* NCFM or *bshB* from *L. johnsonii* NCK88 in *Escherichia coli* C600 helped to significantly increase the colonization biomass of recombinant bacterium in the feces of germ-free mice compared with that of wide-type strain (Dimarzio, 2016). Conversely, BSHs from *L. gasseri* and *Blautia obeum* (Alavi et al., 2020) are even detrimental to the colonization of these strains and other pathogens in the GIT, contradicting their assumed roles (Moser and Savage, 2001). Therefore, the impact of BSH-catalyzed deconjugation on the adhesion and colonization of host strains is context-specific and depends on the BA core, the conjugated amino acid, and the BSHs (Foley et al., 2021). Further investigations are highly warranted to gain new insights into substrate-gene-performance, and to enable the engineering of probiotics to regulate their colonization abilities.

The mechanism underlying the contribution of BSHs to the fitness and colonization of probiotics is yet to be explored, which may include enhancing the adhesion abilities of host strains (Yang et al., 2019). As cell surface hydrophobicity is sometimes correlated with adhesion, BSH-positive *L. plantarum* PYPR1, which showed good surface hydrophobicity, could effectively adhere to epithelial cells and colonize (Yadav et al., 2016). The second mechanism could involve the incorporation of cholesterol moiety into bacterial membranes, which may change their fluidity, permeability, tensile strength and net charge, thus improving the colonization ability and survival of these microbes in the gut by enhancing bile tolerance and sensitivity to host defensins (Taranto et al., 2003). *In vivo* studies have demonstrated that the production of MCBAs positively correlated with the colonization of BSH-active bacteria (Rimal et al., 2024). It has also been shown that BSH activities of intestinal *Bifidobacteria* can increase the hydrogel-forming abilities of certain BAs, which may improve the colonization ability and survival of these bacteria in the GIT (Jarocki and Targoński, 2013; Jarocki et al., 2014). Recent evidence suggests that substrate specificities of BSHs from *L. acidophilus* and *L. gasseri* govern their stereospecific interaction mechanisms’ fitness and host colonization by limiting bacterial death when exposed to GCDCA (Foley et al., 2021), which is another strategy for *Lactobacilli* to adapt to their host niche (O'Flaherty et al., 2018). Besides BSHs, there are several other colonization-associated genes which can be divided into two categories: intestinal tissue-anchored elements and signaling molecules (Duary et al., 2012; Xiao et al., 2021). Thus, BSH-promoted strategy may work alongside with other mechanisms to help microbes to colonize the host GIT.

#### 2.1.3 Nutritional role

Bile acids can be utilized as nutrients, electron acceptors and environmental signals by the intestinal microbiota (Urdaneta and Casadesús, 2017). Amino acids released from BA deconjugation may be further converted into carbon dioxide, ammonium and sulfate, which serve as carbon and nitrogen sources for bacterial sustenance and survival (Tanaka et al., 2000; Ridlon et al., 2016). Huijghebaert and Hofmann (1986) and Van Eldere et al. (1988) found that BSH-positive strains of *Clostridium* utilized taurine as an electron acceptor and their growth rates were increased in the presence of tauroconjugated BAs and taurine. It has also been demonstrated that transcription of the *B. longum bsh* gene is coupled to a homolog of *glnE* that encodes a glutamine synthetase adenyltransferase that forms part of the nitrogen regulation cascade (Tanaka et al., 2000). Based on these findings, it was concluded that BA deconjugation may confer a nutritional advantage to hydrolytic strains. However, the sulfonic moiety of taurine can be dissimilated to hydrogen sulfide which is toxic to both bacteria and human host (Jarocki et al., 2014), which may refute this hypothesis. Consequently, the nutritional roles of BA deconjugation require further investigation.

#### 2.1.4 Bacterial membrane alterations

The fluidity, composition, permeability, hydrophobicity, and net charge of bacterial membranes all determine the extent of damage by host defensins. Since BAs could disorganize the structure of cell membranes to display antimicrobial activity (Bustos et al., 2018), by changing the BA pool, BSH expression regulates gut bacterial membrane integrity and exposure to membrane-damaging BAs (Foley et al., 2021; McMillan et al., 2024), overall membrane composition and host cell internalization (Marchesini et al., 2011). Additionally, BSHs also facilitate the incorporation of BAs or cholesterol into bacterial membranes (Taranto et al., 2003; Bi et al., 2016), which may increase their tensile strength or change their fluidity and net charge, and ultimately improve the colonization and survival of these microbes in the gut (Taranto et al., 2003). Taken together, BSHs can directly and indirectly alter bacterial membranes, but the exact mechanism of membrane disruption likely varies in a substrate-dependent manner (Foley et al., 2021) and needs further extensive investigations.

#### 2.1.5 Ameliorating drug resistance

Over the past few decades, the overuse and misuse of antibiotics have led to alterations in the intestinal microbiota balance and the rapid emergence of antibiotic-resistant pathogens (He et al., 2012). As a global public health concern, antibiotic resistance has become an increasingly serious obstacle in the treatment of infections (Korpela et al., 2016). It has been found that gut microbial BA metabolism influences the solubility and transport of poorly water-soluble drugs (Enright et al., 2018), and BA-activated receptors also play critical roles in the regulation of drug metabolism and detoxification (Li and Chiang, 2014). Under simulated gastric conditions, He et al. showed that deconjugated BAs produced by BSH hydrolysis could destruct cellular membrane permeability of selected foodborne pathogens, thus increasing their antibiotic susceptibility and eventually effectively inhibiting their growth (He et al., 2012). A more recent study demonstrated that use of macrolide in pre-school children could cause decrease in BSH activity and increase in macrolide resistance, also identifying a significant correlation between BSH enzyme and drug resistance (Korpela et al., 2016). Analogously, BSH was reported to be potentially involved in ameliorating multidrug resistance through the generation of unconjugated BAs with the capacity to access and inhibit P-gp ATPase (Enright et al., 2018). However, since BSHs are phylogenetically and structurally related to penicillin acylase, another member of Ntn hydrolase superfamily, some of them (like *L. paragasseri* JCM 5343^T^ BSH) are bifunctional enzymes that degrade both BAs and β-lactam antibiotics (Kusada et al., 2022), thereby conferring the host gut bacteria antibiotic resistance and improving their survival in the mammalian intestine. More *in vivo* studies are therefore needed to decipher the exact roles that BSHs play in drug resistance. Anyway, these findings may provide guidance in the design of effective BSHs-based probiotic-antibiotic combination therapies for the improvement of intestinal microbiota balance.

#### 2.1.6 Signal transduction

Gut microbiome plays a prominent role in signal transduction via transforming primary BAs into secondary BAs by BSH deconjugation. In bacteria, many natural phenomena including bioluminescence, virulence, biofilm formation, and antibiotic production are now known to be regulated through quorum sensing. Acyl homoserine lactones (AHLs) serve as main signal molecules among many gram-negative bacteria, and quorum sensing mediated by these AHL molecules can be quenched by AHL acylases, which has been regarded as a new antimicrobial strategy (Mukherji and Prabhune, 2015). Because AHL acylases and BSHs both belong to the Ntn hydrolase superfamily, they show similar mode of action, and it has been recently demonstrated that BSH enzymes possibly participate in the disruption of quorum sensing and establishment of pathogenesis (Mukherji and Prabhune, 2015). Therefore, BSHs are involved in both pathogenic and probiotic roles, and have been recognized as “niche factors.”

### 2.2 Impacts of microbial BSHs on host metabolism

#### 2.2.1 Regulation of bile acid metabolism

There are now hundreds of known modifications to BAs and thousands of BA-associated genes, which greatly expand the chemical diversity of the BA pool (Mohanty et al., 2024a). Among them, increased BSH activity has been shown to influence BA composition through FXR, a ligand activated transcription factor highly expressed in the liver and intestine which controls hepatic BAs biosynthesis and enterohepatic circulation (Xie et al., 2020; Chiang and Ferrell, 2022). Reduced FXR activity increases the synthesis of BAs from cholesterol via the rate-limiting enzyme CYP7A1 and promotes the downregulation of the small heterodimer partner (SHP) which in turn activates the liver X receptor (LXR) and upregulates the adenosine triphosphate-binding cassette transporters G5 and G8 (ABCG5/8), thus promoting the conversion of cholesterol into BAs (Liu et al., 2021; Chiang and Ferrell, 2022). Most endogenous BAs bind and activate FXR, however, glycoursodeoxycholic acid in humans and tauro-β-muricholic acid in mice serve as FXR antagonists, which could counterbalance the homeostasis of BAs (Joyce et al., 2014a; Xie et al., 2020).

#### 2.2.2 Lowering serum cholesterol levels

As predicted by WHO, cardiovascular diseases (CVDs) will remain the leading cause of death by 2030, affecting approximately 23.6 million people around the world (Ekong, 2021). Hypercholesterolemia is considered as one of the major risk factors for CVDs, and even a small reduction in serum cholesterol of 1% can reduce risk of coronary heart disease by 2–3%. Although pharmaceuticals are available to treat this disease, they are often expensive and suboptimal, and have unwanted side effects. In 1974, Mann and Spoerry (Mann, 1974) observed that the serum cholesterol levels of African Maasai men are lower than normal people after long-term consumption of fermented milk containing probiotics. Since then, oral administration of probiotics has increasingly been reported to have both preventive and therapeutical hypocholesterolemic effects (Jones et al., 2013), some of which can reduce serum cholesterol levels by as much as 22–33% (De Smet et al., 1998). Some *in vivo* clinical trials have also shown that oral consumption of probiotics or products containing them reduce both cholesterol and blood lipid concentrations in hypercholesterolemic subjects, while levels of triglycerides and high-density lipoprotein remain unchanged (Malpeli et al., 2015). Therefore, much effort has been devoted to developing BSH enzymes and BSH-producing probiotics as cholesterol-lowering agents.

With regard to the cholesterol removal effects of probiotics, several possible mechanisms have been proposed (reviewed in Jones et al., 2013; Horáčková et al., 2018): (1) co-precipitation of cholesterol with deconjugated BAs, on the one hand, deconjugated BAs produced by BSH hydrolysis are less-soluble under acidic conditions (pH < 0.6) and co-precipitate with cholesterol; on the other hand, deconjugated BAs cannot be reabsorbed by the intestines and are excreted in the feces, which will increase the *de novo* synthesis of BAs from cholesterol, thus leading to hypocholesterolemic levels (Horáčková et al., 2018); (2) cholesterol assimilation, cells directly bind cholesterol from the media to cellular surface under anaerobic conditions (Widodo et al., 2021), which makes less cholesterol available for absorption. Although several controversies exist, this mechanism has been shown to correlate with BSH activity and the parameters of bile resistance (Aswal et al., 2023); (3) synergistic effects of co-precipitation and bacterial assimilation (Kumar et al., 2012); (4) incorporation of cholesterol into cellular membrane (Miremadi et al., 2014), because co-precipitation of cholesterol with deconjugated BAs cannot occur *in vivo* since the intestinal pH is usually higher than 6.0 (Reis et al., 2017), probiotics may rely on this mechanism to remove cholesterol; (5) other mechanisms: conversion of cholesterol into coprostanol by cholesterol reductase of the strains (Horáčková et al., 2018), inhibition of Niemann-Pick C1-like 1 (NPCL1) protein (Jones et al., 2013), and production of short chain fatty acids, prebiotics that inhibit hepatic cholesterol synthesis (Ashaolu et al., 2021).

However, most of the hypotheses raised to date are based on *in vitro* experiments, and few attempts have been made to evaluate the possible hypocholesterolaemic mechanisms based on *in vivo* trials. Furthermore, the cholesterol-lowering efficacy of probiotics are influenced by many factors, such as the concentration of BAs, strains used and viable count, which complicates the elucidation of the exact mechanism underlying. But modification of BA metabolism through BSH activity is considered to be the core mechanism of the anti-cholesterolaemic effects (Agolino et al., 2024). To date, numerous studies have linked BSHs to hypercholesterolemia in mice and humans (reviewed in Jones et al., 2013; Reis et al., 2017). De Smet et al. were the first to explicitly demonstrate the efficacy of a BSH-active probiotic for lowering low-density lipoprotein (LDL) cholesterol levels in an animal model by increasing fecal BAs output (De Smet et al., 1998), which is the major pathway to remove cholesterol (Jones et al., 2012a). Subsequent trials in humans and mice have reported similar effects from oral administration of BSH-producing *Lactobacilli* strains (Jones et al., 2012a; Jones et al., 2012b; Wang et al., 2019) and immobilized BSH enzyme from *L. buchneri* ATCC 4005 (Sridevi et al., 2009). Importantly, the cholesterol-lowering effects of many BSH-positive *Lactiplantibacillus* strains were related to increased hepatic BA synthesis mediated by the FXR signaling pathway via upregulation of hepatic CYP7A1 in mice (Wang et al., 2019; Liu et al., 2021; Zhu et al., 2022; Zhao et al., 2024). More recently, follow-up trials reported that changes in BA composition and cholesterol synthesis were correlated with fibroblast growth factor 19 (FGF19, a key mediator of FXR signaling) in *L. reuteri* (Martoni et al., 2015), and gut-liver FXR-FGF19 axis in *L. delbrueckii* (Hou et al., 2020). BSH enzymes with different substrate specificities can modulate bile acid profiles differently and, consequently, have different mechanisms for cholesterol removal (Agolino et al., 2024). For instance, a BSH mutant F67A, which exclusively hydrolyzed TCA, reduced serum cholesterol levels in high-fat diet (HFD) mice through the modulation of intestinal FXR pathway, while another mutant YB81 preferred to hydrolyze GCA and reduced cholesterol levels by regulating changes in the intestinal flora and BSHs within the flora (Zhao et al., 2024). Therefore, mechanisms underlying the cholesterol-lowering effects of BSHs *in vivo* include elevated fecal excretion of deconjugated BAs, inhibition of hepatic cholesterol synthesis via FXR signaling, and induction of cellular LDL cholesterol uptake (Jones et al., 2012a). In addition to BSHs, gastrointestinal cholesterol efflux system encoded by ABCG5/8 (Jones et al., 2013) and S-layer protein (Guo et al., 2018) also contribute to the hypocholesterolemic activity of BSH-active probiotics.

#### 2.2.3 Reducing cardiovascular risk

CVDs are a group of disorders of the heart and blood vessels, and their evolution and progression depend on multiple factors, like hypercholesterolemia, dyslipidemia, diabetes mellitus, hypertension, smoking, etc. Among them, hypercholesterolemia caused by elevated levels of total and LDL cholesterol constitutes an important cardiovascular risk factor (Neverovskyi et al., 2020). Oral administration of *L. plantarum* 299v has already been shown to reduce CVD risk factors in smokers (Naruszewicz et al., 2002). However, there are limiting data regarding the clear-cut effects of BSHs on host cardiovascular risk. Recently, several *in vivo* studies with human beings have found that the total relative activity of gut bacterial BSH is negatively correlated with cardiovascular risk, total and LDL cholesterol levels, as well as the risk of dyslipidemia (Jia et al., 2020; Neverovskyi et al., 2020). The deconjugated BAs produced by BSH hydrolysis can regulate cardiovascular function through nuclear receptors (FXR, PXR and LXR) and G-protein-coupled receptors (TGR5 and muscarinic receptors) (Zhang et al., 2024).

#### 2.2.4 Regulation of host lipid and energy metabolism

Since deconjugated BAs are less efficient than their conjugated counterparts in the emulsification of dietary lipids, BSHs may cause disorders of lipid digestion in the hosts, thus leading to weight loss of the host (Huang et al., 2019). Numerous *in vitro*, *in vivo* and clinical trials have pointed to the fact that BSH-encoding strains and BSH enzymes directly reduced fat absorption, thus serving as functional foods for the treatment of obesity. An *in vitro* study found that BSH-producing *L. plantarum* AR113 and *L. casei* pWQH01 decreased hepatic lipid accumulation, while BSH-negative *L. casei* LC2W did not exert this beneficial effect (Huang et al., 2020). In mice, rats and hamsters, oral consumption of BSH enzymes, including recombinant BSH (Spratt et al., 2014), as well as BSH-active probiotics like *L. plantarum* H-87 (Liang et al., 2021) and *L. acidophilus* GOLDGUT-LA100 (Zheng et al., 2024) could inhibit their liver fat deposition, lipid digestion and body weight, eventually alleviating HFD-induced obesity. Using a controlled experimental system, Joyce et al. reported *in vivo* in animal models that increased BSH expression in the gut ecosystem led to lower body weight gain, lower adiposity and reduction of both circulating serum cholesterol and liver triglycerides (Joyce et al., 2014a). A systematic review and meta-analysis of randomized clinical trials showed that administration of probiotics counteracted some aspects of dislipidemia in hypercholesterolemic patients (Jiang et al., 2020). These findings ultimately suggest that BSH enzyme is a potential target for weight regulation and could be applied in the design of intervention strategies in humans and the agricultural sector. However, direct evidence supporting the significant role of intestinal BSHs in host lipid metabolism, energy harvest, as well as body weight change is still very limited. It has been shown that BSH from *L. johnsonii* LB1 (Dimarzio, 2016) does not affect the weight gain of mice, whereas BSH-deleted *B. thetaiotaomicron* causes significant weight loss in mice (Yao et al., 2018). Besides, the antioxidant tempol (Li et al., 2013), an Atlantic brown algae extract (Huebbe et al., 2017), theabrownin from Pu-erh tea (Kuang et al., 2020), and resistant starch (Li et al., 2024), which could reduce the BSH activities of gut microbiome, also remarkably ameliorate diet-induced obesity in mice or humans. These inconsistent findings thus call for more in-depth studies to decipher the BSH-mediated anti-obesity effect since it is more complex and influenced by many factors, such as diet, host, BSH activity, and bile acids.

The mechanism underlying weight regulation of microbial BSHs needs to be further elucidated. It has been reported that BSH activity in the gut regulates host lipid metabolism by impacting the expression of genes involved in cholesterol transport as well as lipid transport and synthesis in the duodenum, ileum and liver, such as *Ppary* and *Angptl4* (Joyce et al., 2014a), and the fasting-induced adipose factor (*FIAF*) (Kersten, 2023). Some recent studies suggest that BSH activity alters gut microbiota and reduces obesity in mice through an FXR-mediated mechanism (Li et al., 2013; Liang et al., 2021), while unconjugated BAs affect TGR5-mediated adipose tissue development and weight loss (Svensson et al., 2013), and that there is a subtle interplay between FXR and TGR5 that warrants further investigations. Besides, BSH activity and unconjugated BAs also regulate the expression patterns of host circadian rhythm (e.g., *Dbp*) and other genes central to circadian clock (Joyce et al., 2014a; Joyce et al., 2014b; Govindarajan et al., 2016), which is clearly related to alterations in weight regulation and energy metabolism (Boege et al., 2021), and its disturbance causes the pathogenesis of obesity, inflammatory bowel diseases (IBD) and several liver diseases (Li and Chiang, 2014). Notably, obesity development is a complex physiological issue, and BSH-mediated bile salt metabolism is only one of several potential mechanisms by which microbiota affect host weight gain and energy harvest (Joyce et al., 2014a). Direct and controlled approaches are therefore required to obtain complete understanding of BSH-mediated regulation of host lipid metabolism and weight gain.

#### 2.2.5 Disruption of normal intestinal conditions

Due to the altered bile acid profile and abnormal lipid metabolism, deconjugation of bile salts by BSHs results in disruption of normal intestinal conditions and development of many intestinal diseases, such as short bowel syndrome. It has also been proposed that unconjugated BAs produced by BSHs are further modified into DCA, LCA and other secondary BAs by bacterial 7α-dehydroxylase (Figure 1), which may promote colorectal cancer, cause DNA damage and reactive oxygen species-associated oxidative stress in epithelial intestinal cells, or lead to impaired colonic mucosal function that would cause diarrhea, inflammation, and some other GIT diseases (Sun and Kato, 2016). Compared to its primary counterpart, DCA exhibits a 10-times higher antimicrobial activity, thus inhibiting the growth of many bacteria and altering gut microbial profiles (Liong et al., 2015). More hydrophobic secondary BAs also promote cholesterol crystallization, and the crystallized cholesterol together with bile pigments and calcium salts form gallstones (Thamer, 2022). Fortunately, the most commonly used probiotics (*Lactobacilli* and *Bifidobacteria*) cannot dehydroxylize deconjugated BAs, so they will mainly be precipitated and eliminated from body via feces, indicating no safety concern associated with these strains (Ahn et al., 2003). In contrast to these detrimental effects of BSHs on normal intestinal conditions, recent research indicated that *B. fragilis* BSH can alleviate necrotizing enterocolitis via restoring gut microbiota dysbiosis and BA metabolism balance (Chen et al., 2024), which necessitate an intense study into gut microbial BSHs to better understand their roles in the pathogenesis and treatment of intestinal disorders.

#### 2.2.6 Modulation of local immune homeostasis

BSH-mediated deconjugated BAs are recognized by host-encoded receptors as signaling molecules to regulate host immunity, since they can modulate a variety of intestinal effectors, including the dendritic cell differentiation and inducible nitric oxide synthase (Joyce et al., 2014b), and antimicrobial peptide RegIIIγ produced by intestinal paneth cells (Joyce et al., 2014a). An *in vivo* study showed that BSH-active strain *L. mucosae* DPC 6426 could modulate the immune system of mouse (Ryan et al., 2019). However, little information is available with regard to the effects of BSHs on regulating immune homeostasis, and further studies in this direction are necessary.

#### 2.2.7 Correlation with liver diseases

BA homesostasis modulates glucose and lipid metabolism via gut-liver axis and gut-adipose axis, respectively, and dysregulation of BA metabolism and circadian disturbance cause the pathogenesis of liver metabolic diseases including steatosis, type 2 diabetes, obesity, liver cirrhosis, hepatic cancer and non-alcoholic fatty liver disease (NAFLD) (Jia et al., 2020; Bourgin et al., 2021; Chiang and Ferrell, 2022). It has been shown that BSH enzymes participate in the pathogenesis of many liver diseases, including NAFLD, cholestasis, and colorectal cancer. In NAFLD patients, total fecal BA concentrations are generally elevated, whereas suppression of bacterial BSH activity by its inhibitor caffeic acid phenethyl ester can alleviate NAFLD through inhibiting intestinal FXR signaling (Zhong et al., 2023). Supplementation of BSH-producing *L. plantarum* Y15 could effectively ameliorate liver histopathological changes in mice, including relatively weak fat damage and unobserved small focal necrosis (Liu et al., 2021). Cholestasis refers to impaired bile flow from the liver to the intestine, and it significantly reduces BSH gene abundance and enzymatic activity in preterm neonates, indicating the requirement of this enzyme in early neonatal development (Lynch et al., 2023). Recently, it has been demonstrated that ursodeoxycholic acid, which is commonly used to treat cholestasis, enriches intestinal BSH-producing *Bacteroidetes* in intrahepatic cholestatic pregnancy (Ovadia et al., 2020). Therefore, BSH-active *Bacteroidetes* coupled with ursodeoxycholic acid can be used to cure this disease. As liver tumorigenesis is suppressed through an immune pathway which is stimulated by gut-derived primary BAs, but not secondary BAs (Ma et al., 2018), the overexpression of the *bsh* gene from *B. fragilis* NCTC 9343 in *B. fragilis* 638R, which led to the increase of unconjugated BAs in the colon, accelerated the progression of colorectal cancer under HFD treatment (Sun L. et al., 2023). Thus, modulating BSH activity to limit secondary BA metabolism would be a promising therapeutic for the prevention and treatment of colorectal and hepatic cancers.

#### 2.2.8 Neuroprotective effect

As key modulators of the microbiota-gut-brain axis, BAs can enter the systematic circulation and across the blood–brain barriers, and subsequently display neuroprotective potential against several neurological diseases by targeting endogenous receptors, such as Alzheimer’s disease (Wu et al., 2024), major depressive disorder (Sun et al., 2022), etc. Besides, NAFLD also affects the development of neurological diseases via BA signaling, while inflammatory and systematic metabolic disorders in the brain are regulated by FXR and TGR5 (Ren et al., 2022). Recently, it has been found that the endogenous bile acid, tauroursodeoxycholic acid, may be an effective therapeutic to prevent and treat Alzheimer’s disease (Song et al., 2024). Besides, CDCA also exerts antidepressant effect through FXR signaling and it has become a biomarker and target potentially important for the diagnosis and treatment of major depressive disorder (Li et al., 2023). Therefore, BSHs may be linked to neurological diseases, and administration of BSH-active probiotics and BSH enzymes would become novel therapies to treat these neurological disorders by targeting gut microbiota.

## 3 Potential applications of BSHs and BSH-active probiotics

### 3.1 Serve as selection and diagnostic biomarkers

Generally, BSH activity has been widely recognized as a functional probiotic biomarker due to its beneficial effects on GIT microbiota and host health, such as microbial bile tolerance, antimicrobial activity, colonization in the GIT, and cholesterol reduction (Miremadi et al., 2014). Bile salt hydrolase gene has been used as a potential food-grade selection marker for the construction of novel vectors for lactic acid bacteria (Yin et al., 2011). The TG motifs of BSH B and C from *L. johnsonii* PF01 can enhance the promoter strength, which makes them good candidate promoters for the construction of an *E. coli*-*Lactobacilli* shuttle vector (Chae et al., 2013). BSH-encoding genes also serve as valuable molecular markers for phylogenetic studies, and specific identification and selection of probiotics (Jarocki and Targoński, 2013), as well as detecting the contamination of *E. faecalis* in fermented foods (Yoon et al., 2020).

Gut microbiota-derived BSH activity act as a serum metabolomic biomarker, and its changes have been linked to many human diseases, such as hepatobiliary disease (Parasar et al., 2019), Crohn’s disease (Wang Y. et al., 2021), and non-obese liver fibrosis (Lee et al., 2020). Recently, chemoproteomic tools (Parasar et al., 2019), the non-invasive diagnostic tool (Khodakivskyi et al., 2021), and several BSH activity-based probes (Han et al., 2024) have been developed to quickly and cost-effectively assess and quantify BSH activity across a broad range of biological settings including pure enzymes and bacteria, intact fecal slurries, noninvasive imaging in live animals, and the entire gastrointestinal tract of mice and humans, which facilitate the prediction and diagnosis of the clinical status of these diseases. Taken together, BSHs can be used as biomarkers for the construction of food-grade vectors, selection of probiotics and medical diagnosis.

### 3.2 Development of biotherapeutic agents

#### 3.2.1 Hypocholesterolemic agent development

Partly due to their cholesterol-lowering effects through enterohepatic axis regulation, BSH-producing probiotics have now been largely used as hypocholesterolemic food supplements or “drugs” in this day and age (Palaniyandi et al., 2020). A growing number of people with hypercholesterolemia have used these products containing so-called “friendly” bacteria. In 2012, Cardioviva ™ (containing *L. reuteri* NCIMB 30242) was launched, the first and only probiotic on the market that has been clinically proven to naturally reduce total and LDL cholesterol levels by 9 and 11.6% in hypercholesterolaemic adults, respectively (Jones et al., 2012a; Jones et al., 2012b). More recently, administration of probiotic capsules containing the BSH-active *L. plantarum* ECGC 13110402 (LP_LDL_^®^), a commercialized probiotic bacterium, could significantly reduce total cholesterol levels in hypercholesterolemic subjects compared to the placebo group (Keleszade et al., 2022). In the future, more biotherapeutic agents, including fermented foods and additives of healthy diets such as yogurt harboring BSH-active *Lactobacilli*, will be developed to overcome hypercholesteremia.

#### 3.2.2 Biotherapeutics for the treatment of infectious diseases

*Giardia duodenalis* (also known as *G. lamblia* or *G. intestinalis*) is responsible for giardiasis, one of the most common and widely spread intestinal parasitic diseases worldwide, affecting both humans and animals. The spread of resistant parasite strains and the lack of appropriate medications urgently call for the development of novel therapeutic strategies. Recent findings suggested that BSH from the probiotic strain *L. johnsonii* La1 exhibited anti-giardial activities both *in vitro* and *in vivo* (Allain et al., 2017), and BSH activity has been included among the selection criteria for identifying anti-*Giardia Lactobacilli* strains. This effect is related to the generation of deconjugated BAs, which are toxic to the parasite in a dose-dependent manner, thus displaying a significant deleterious effect on the parasite. In another work, the anti-giardial effects of various *Lactiplantibacillus* strains were tested, and BSH-producing *L. johnsonii* La1 and *L. gasseri* CNCM I-4884 showed anti-parasitic abilities *in vitro* (Allain et al., 2018). However, *in vivo* studies demonstrated that *L. gasseri* CNCM I-4884 other than *L. johnsonii* La1 could significantly reduce *G. duodenalis* infection in a suckling mice model (Allain et al., 2018). Therefore, further experiments are highly warranted to investigate the potential of BSHs in treating giardiasis and the possible mechanisms underlying. Knowledge gained here represents a step toward the development of new prophylactic strategies to combat *G. duodenalis* in both humans and animals.

*Clostridium difficile* infection (CDI), which is caused by gut microbiota dysbiosis due to consumption of antibiotics, is one of the most common nosocomial gastrointestinal infections. CDI includes a broad range of disorders ranging from diarrhea to colitis and toxic megacolon, and their incidence, severity and costs are continuously increasing (Bourgin et al., 2021). The germination process of this bacterium is mediated by a host-derived molecule including BA sensing through the germinant receptor (Zhu et al., 2018). Matthew et al. found that BSHs with varying substrate preferences restricted *C. difficile* spore germination and growth *in vitro*, and colonization in pre-clinical *in vivo* models (Foley et al., 2023). Besides, *B. ovatus* SNUG 40239 also inhibit the growth of *C. difficile* by its BSH activity *in vitro* (Yoon et al., 2017). *In vitro* studies also showed that BSH-mediated hydrolysis of TCA effectively suppressed *C. difficile* germination, and administration of *E. coli* cells expressing highly active BSH contributed to the efficacy of fecal microbial transplantation in the treatment of CDI (Mullish et al., 2019). These results will facilitate the development of novel preventative or bacteriotherapy strategies targeting gut microbial BSHs to cure CDI. However, *C. difficile* also contains a BSH enzyme which favors taurine-conjugated BAs (Aguirre et al., 2022), highlighting the need for greater understanding the function of bacterial BSHs in the treatment of CDI.

IBD is a chronic inflammatory condition of the GIT characterized by a dysregulation of the gut mucosal immune functions and a dysbiotic gut microbiota occurring in genetically susceptible hosts. It encompasses two major phenotypes: ulcerative colitis and Crohn’s disease. Patients affected by IBD display low levels of gut microbiome-associated BSH activity (Jia et al., 2020), and many BSH-producing probiotics have been used as dietary supplements or drugs for the management of IBD (Gadaleta et al., 2022). *In vivo* studies showed that the probiotic candidate strain *B. dorei* BDX-01 (Sun X. et al., 2023), and BSH eznymes from *L. plantarum* AR113 (Feng et al., 2023) could ameliorate dextran sulfate sodium induced colitis in mice by regulating intestinal BSH activity and the FXR-NLRP3 signaling pathway, and antibiotic treatment could not abolish the protective effect of the former (Sun X. et al., 2023).

### 3.3 BSH enhancers and inhibitors development

#### 3.3.1 BSH enhancers development

Obesity, one of the main causes of metabolic syndromes, is usually reflected as body fat accumulation, liver abnormalities, dyslipidemia and insulin resistance. It is also a group of inter-related metabolic conditions that greatly increase the risk of developing CVDs, diabetes and cancers (Gil-Rodríguez and Beresford, 2021). Nowadays, obesity has become a worldwide health problem, creating an urgent demand for its effective prevention and treatment. Although factors, such as diet and genetic background, contribute significantly to the prevalence of obesity, many studies have linked the gut microbiome to obesity and weight gain in humans and animals (Joyce et al., 2014b). Consequently, manipulation of gut microbiota might be a promising strategy to control obesity.

As unconjugated BAs are less efficient than conjugated molecules in the emulsification of dietary lipid and the formation of micelles (Huang et al., 2019), BSH enzymes and BSH-enriched microbiota are negatively related to the body weight gain and energy storage of the hosts (Korpela et al., 2016; Liang et al., 2021). Enhanced *in situ* BSH activity of gut microbiome not only causes weight loss in conventionally raised mice (Joyce et al., 2014a), but also lowers serum cholesterol levels in humans (Jones et al., 2008). Therefore, BSH enhancers have become one of the key mechanisms in the anti-hyperlipidemic and anti-hypercholesterolemic effects and have a potential application in human health. Thus, enhancing gut microbe-enriched BSH activity by dietary supplementation of highly BSH-active probiotics and BSH enhancers may offer potential as a biological alternative to pharmaceutical interventions to prevent and treat obesity and hypercholesterolemia.

#### 3.3.2 BSH inhibitors development

Antibiotic growth promoters (AGPs) are a group of antibiotics used at subtherapeutic levels to improve the average daily weight gain and feed conversion efficiency in agricultural animals (Ayalew et al., 2022). Since the 1950s, AGPs have been successfully used by agricultural animal producers to improve growth performance. However, recent epidemiological studies strongly indicated that long-term and improper uses of AGPs resulted in the emergence of antimicrobial resistance and antibiotic residues in animal products, leading to food safety and public health threats (Ayalew et al., 2022). Consequently, there is a global trend to ban the use of AGPs in animal production, which necessitates the need to develop effective non-antibiotic alternatives to improve animal performance.

Numerous studies have shown that the growth-promoting effects of AGPs are inversely correlated with the decreased BSH activities of gut microbes (Smith et al., 2014; Rani et al., 2017) or the reduced abundance of BSH-producing bacteria in the small intestine (Robinson et al., 2019). As previously described, deconjugation of BAs by gut microbiota-derived BSHs results in lipid malabsorption and attenuates energy harvest, which may cause weight loss of the hosts (Joyce et al., 2014a; Korpela et al., 2016; Liang et al., 2021). Conversely, inhibiting the activities of intestinal BSHs can enhance lipid metabolism and energy harvest, thus improving the growth performance and feed efficiency of food animals (Bustos et al., 2018). Therefore, specific BSH inhibitors have been proposed as promising alternatives to AGPs for fattening food animals.

To date, various types of BSH inhibitors have been developed as novel alternatives to AGPs to enhance the productivity and sustainability of food animals. As conventional BSH inhibitors, high concentrations of cooper (CuCl_2_) and zinc (ZnSO_4_) have been reported to promote food digestion and body weight gain in different animal models (Liu et al., 2018). But long-term use of these metal ions will increase their accumulation in treated animals and the environment. Iodine reagents, such as sodium periodate, sodium iodate and potassium iodate, could also strongly inactivate BSHs (Smith, 2013), but their safety remains to be determined.

To discover more potent BSH inhibitors with satisfying safety profiles, many efforts have been devoted to screening from both natural and synthetic compounds. Several natural plant extracts, such as 2α-OH-protopanoxadiol (Xie et al., 2020), amentoflavone (Li et al., 2022a), licochalcone C and isobavachalcone (Li et al., 2022b), as well as green tea and *B. vulgaris* root extracts (Dibamehr et al., 2021) exhibit inhibitory effects against BSH enzymes or BSH-producing bacteria both *in vitro* and *in vivo*, thus serving as potential alternatives to AGPs. Using a high-throughput screening method, Smith et al. identified several potent inhibitors of *L. sali*var*ius* NRRL B-30514 BSH from 2,240 various compounds, including riboflavin, caffeic acid phenethyl ester and carnosic acid (Smith et al., 2014). Thereafter, efficacies of these three inhibitors were evaluated using a chicken model (Geng et al., 2020). Among them, riboflavin and caffeic acid phenethyl ester could also inhibit the activities of BSHs from *L. acidophilus* PF01 (Lin et al., 2014) and *L. gasseri* FR4 (Rani et al., 2017) which have significantly different protein sequences and substrate spectrum. Besides, riboflavin has already been approved by the FDA of United States of America for the use as a feed additive to overcome vitamin B2 deficiencies in animals (Smith et al., 2014), and this compound alone or in conjunction with *L. gasseri* FR4 could also enhance the body weight of pigs and feed efficiency (Rani et al., 2017). Actually, there are many different subtypes of gut microbial BSHs with various substrate specificities (O'Flaherty et al., 2018), but the inhibitors obtained exhibit either moderate inhibitory or no inhibitory activities toward other BSHs, which greatly limits their practical usages.

Although BSH protein sequences vary largely among different gut strains, all BSHs possess six highly conserved active-site residues that include the catalytic cysteine (Cys2) (Dong and Lee, 2018). Based on this structural conservation, Adhikari et al. developed a covalent pan-inhibitor GR-7 which targeted both *B. longum* and *B. thetaiotaomicron* BSHs in a dose-dependent manner (Adhikari et al., 2020). This compound could effectively inhibit the deconjugation of BAs *in vitro* and *in vivo*, and did not significantly affect the viability of gut bacteria. To reduce off-target effects and enhance the potency and gut permeability, a LCA, the second-generation gut-restricted pan-BSH inhibitor, has been developed. As compared with the first generation compound, it displayed reduced toxicity toward mammalian cells and suppressed BSH activities in complex biological samples, including purified samples, bacterial cultures and conventional mouse fecal slurries (Adhikari et al., 2021). The usage of this inhibitor also prevented the development of hepatic inflammation and pathologic intestinal permeability in rats fed a choline-deficient, L-amino acid-defined HFD (Li et al., 2022c). Improved knowledge in the roles of BSHs and BSH-producing bacteria are highly warranted to design rational tailored pan-BSH inhibitors that would enhance animal health and performance. BSH pan-inhibitors could also be cooperated with certain BSH-positive probiotics to maximize the beneficial effects of these probiotics by alleviating their potential negative impacts on host fat digestion. Since noncovalent BSH inhibitors can avoid the development of resistance mutations (Adhikari et al., 2020), in the long term, they may prove to be the most effective tool for *in vivo* use. Therefore, further studies may be performed to confirm their growth-promoting effects.

### 3.4 Other applications

Gut bacterial BSH has become a key target for the manipulation of gut microbiome and host health. Targeting this enzyme by dietary supplements, including crude extract, polysaccharides, phenols, saponins, alkaloids and dietary fiber, have been shown to effectively regulate gut microbiota and BAs-FXR signaling, thus modulating human health (Kastl et al., 2022). By modulating BSH activity, several natural products, like L-theanine, capsaicin and epigallocatechin-3-gallate, have been shown to reduce glucose levels (He et al., 2024), offering novel insights and strategies for type 2 diabetes prevention and treatment. Similar results have also been observed in medical constituents, such as caffeic acid phenethyl ester (Gonzalez et al., 2016) and gentamicin (Ma et al., 2023). In addition, recombinant BSH from *B. longum* could enhance the inhibition efficiency of TDCA toward *C. perfringens* virulence in chickens (Alenezi et al., 2024).

## 4 Conclusions and future prospects

Many recent observations have indicated that gut microbial BSHs are involved in a multifaceted array of roles, directly or indirectly in the host and microbial physiologies, thus mediating a gut microbe-host dialog. It has been increasingly shown that BSH activity can reshape the landscape of BAs, which not only influences bacterial physiology, but also regulates a wide range of physiological processes in the hosts. As key gatekeepers and mediators of BA transformation, BSHs have been currently investigated as promising target enzymes in the manipulation of gut microbiota to benefit human and animal health. Although much effort has been undertaken to elucidate the physiological functions of gut microbiota-derived BSHs, detailed studies are still required to uncover their significance more clearly. Upcoming researches of BSHs should focus on their specific roles in the gut microbiome, and beneficial or detrimental effects on the host.

Microbial BSH activity has been considered as a beneficial property that may find importance for novel preventive and therapeutic strategies for conditions associated with BA dysbiosis. Despite the claimed benefits from human clinical studies carried out for the last few decades, controversies still exist in the roles of BSHs in bile detoxification, bacterial colonization, ameliorating drug resistance, lowering serum cholesterol levels, regulation of lipid metabolism and treatment of metabolic syndromes, owing to the fact that different clinical and methodological trials have complicated the use of probiotics in reaching a decisive outcome. Therefore, more properly-designed *in vivo* clinical trials are urgently warranted to investigate the exact function of BSHs to clarify conflicting findings in these fields and to explore the precise underlying mechanisms to have better formulations for animal and human consumption.

The substrate preferences of microbial BSHs have been extensively reviewed (Dong and Lee, 2018). Based on their kinetic parameters and specific activities toward various substrates, most BSH enzymes prefer to hydrolyze glycol-conjugated BAs, like GCA, GDCA and GCDCA, a few BSHs show high affinity for tauro-conjugates (TCA, TDCA and TCDCA), whereas others hydrolyze both glycol- and tauro-conjugated BAs. Although many structural analyses and amino acid substitution mutagenesis have been performed, the precise substrate recognition mechanism and the structural basis for the substrate preferences of BSHs remain to be elucidated. As summarized in this review, differences in the substrate specificity of BSHs not only influence the bile-detoxifying effects, colonization ability and growth of gut microbiota, but also affect host physiology, such as lowering serum cholesterol levels and modulating CDI. Overall, substrate preferences may underlie the protective vs. pathogenic effects of BSHs in the animal and human GIT. However, it is still unclear how differences in BSH substrate specificities affect BA pool, gut microbiota and host responses. In-depth analysis of the relationship between the functions and substrate specificities of BSHs would deepen our understanding of the BSH-mediated gut microbe-host dialog, and lay a solid foundation for the rational design of next generation BSH-active probiotics with improved colonization efficacy and persistence in the host as well as their usage as BA-altering biotherapeutics in the treatment of host specific metabolic diseases.

Extensive studies on gut microbiota have suggested that BSHs are key mechanistic microbiome targets for the development of BSH enhancers and inhibitors, which are promising measures for the control of diet-induced obesity in humans and novel non-antibiotic growth promoters in farm animals, respectively. To date, although a number of BSHs have been identified from different commensal bacteria, our understanding of the BSH structure–function relationship is still limited, since BSHs of various origins have different crystal structures and substrate specificities, and some strains even have more than one BSH homolog with different substrate preferences. With the development of artificial intelligence, structural biology, protein engineering, high throughput screening system and bioinformatics, structure-based computations would not only improve our understanding of the catalytic and substrate recognition mechanisms of BSHs, but also facilitate the rational design of BSH enhancers-based weight-reducing aids or BSH inhibitors-based alternatives to AGPs. Future research in this area would be really beneficial for human and animal health.

Although the therapeutic potential of gut microbiota-associated BSHs has been proposed, there are still knowledge gaps regarding the utility of BSHs as promoters of probiotic colonization and as treatments for diseases in human, which have hampered their development. Further studies are needed to better understand the function of BSHs in regulating host metabolism to develop BSH-based biotherapeutics. Extensive advances in the new “omics” technologies (e.g., functional metagenomics, the integrative microbiome-host transcriptome, the integrated transcriptomic and proteomic analyses) and systems biology approaches have allowed us to unravel the complex interactions between gut microbiome, BAs, BSH activity, the liver, GIT and host health. With the development of chemoproteomic tools and quantitative optical readout-based methods, we could also quickly and cost-effectively quantify BSH activity in complex biological samples, thus improving our understanding of altered BSH activities on important physiological processes and diseases. Thereafter, BSH-positive probiotics can be engineered by genome-editing tools such as CRISPR-Cas to be employed as biotherapeuticals for different needs.
